# The effect of T cells on the lncRNA4.9-TGF-β1 axis in HCMV latently infected cells

**DOI:** 10.1097/MD.0000000000042400

**Published:** 2025-05-16

**Authors:** Xiaolian Yi, Lifang Liu, Ran Tao, Wei Li, Huamei Li, Lin He, Yujie Liu, Shiqiang Shang

**Affiliations:** a Department of Laboratory Center, Children’s Hospital Affiliated to Zhejiang University School of Medicine, Hangzhou, China.

**Keywords:** cytokines, human cytomegalovirus, latent infection, lncRNA4.9

## Abstract

Human cytomegalovirus (HCMV), a prevalent double-stranded DNA virus, exhibits a high infectioraten, yet the mechanisms underlying latent infection and activation remain unclear. Viral cyclic reactivation in healthy HCMV and latent infection is usually well controlled by the T-cell response. Long noncoding RNA (lncRNA) is known to play vital roles in physiological and pathological processes. This study investigates the impact of T cells on the expression of lncRNA4.9, transforming growth factor-β (TGF-β), and multiple cytokines during HCMV latent infection. We established an HCMV latent infection model, coculturing human acute monocytic leukemia cell line (THP-1) cells with T cells subjected to different treatments: NC1 (THP-1 cells cocultured with untreated T cells), NC2 (HCMV latently infected group without T cells), phytohemagglutinin A (PHA) group (PHA-activated T cells added), FK506 group (FK506-suppressed T cells added), and T-cell group (untreated T cells added). Cytokines were assessed in cell culture supernatants collected at 24, 48, and 72 hours. Reverse transcription-quantitative polymerase chain reaction examined changes in RNA and HCMV DNA copy numbers after 3 and 5 days. In the HCMV latent infection model, PHA group, T-cell group, and FK506 group exhibited significantly increased interleukin (IL)-6, IL-10, and tumor necrosis factor-alpha secretion. Expressions of lncRNA4.9 and TGF-β1 significantly increased in T-cell group after 3 and 5 days. Expressions of lncRNA4.9 and TGF-β1 significantly decreased in the PHA group after 5 days. DNA copy numbers of HCMV decreased in T cell and PHA groups after 3 days, with no significant change after 5 days. This study reveals that PHA-activated T cells downregulate the expression of lncRNA4.9 and TGF-β1 in HCMV, highlighting the effect of T cells on the lncRNA4.9-TGF-β1 axis during HCMV latent infection. Regardless of T-cell activation, the study also indicates that IL-6, IL-10, and tumor necrosis factor-alpha levels increase during HCMV latent infection.

## 1. Introduction

Human cytomegalovirus (HCMV) is a ubiquitous β-herpesvirus with a population prevalence of 60% to 90%.^[1]^ The expression of immediate-early protein 2 (IE2), encoded by *UL122*, increases during HCMV proliferative infection but significantly decreases after 2 to 3 days and remains low or absent during latent infection.^[2,3]^ When IE2 expression is low or absent, but the *UL138* gene is expressed, it indicates that HCMV is in a latent state. Notably, UL138 serves as a marker protein for HCMV latent infection.^[4–6]^ Long-stranded noncoding RNA (lncRNA) is a class of noncoding RNAs which is longer than 200 nucleotides (nt) and do not encode proteins.^[7]^ Currently, the known mechanisms of action of lncRNAs are 2-fold: regulation at the transcriptional level and regulation at the posttranscriptional level. During the proliferative infection process, HCMV primarily expresses 4 lncRNAs – lncRNA1.2, lncRNA2.7, lncRNA4.9, and lncRNA5.0.^[2,8]^ lncRNA4.9 has been identified from the HCMV expression profile, with a transcript length of 4922 bp.^[9]^ It has been suggested that lncRNA4.9 plays a crucial role in viral DNA replication and proliferation.^[10]^ It has been found that the inhibition of lncRNA4.9 during late latent infection increases IE2 expression and HCMV copy numbers, suggesting activation of HCMV proliferative infection.^[11–13]^ Our research team’s experiments have shown that the inhibition of lncRNA4.9 and NGFI-A binding protein 2 significantly upregulates the expression of early growth response gene 1 (*EGR1*).^[13]^ Previous studies have shown that EGR1 promotes the expression of transforming growth factor-β (TGF-β) after binding with and being activated by IE2.^[14,15]^ There is a correlation between the expression of lncRNA4.9 and TGF-β in HCMV infection. TGF-β is an immunosuppressive factor that inhibits T-cell activation as well as the expression and secretion of several of their cytokines, including interleukin (IL)-2, IL-10, tumor necrosis factor-alpha (TNF-α), and interferon-γ (IFN-γ).^[16]^ This study investigates the impact of T-cell activation on the expression levels of HCMV lncRNA4.9, TGF-β, and various cytokines (IL-2, IL-6, IL-10, TNF-α, IFN-γ, etc) during HCMV latent infection. The findings will provide experimental evidence to support clinical strategies for the treatment and prevention of HCMV.

## 2. Materials and methods

### 2.1. Cell and virus culture

Human acute monocytic leukemia cell line (THP-1) cells (3101HUMTCHu57, mycoplasma-free, 80% confluency) and human embryo fibroblast (HEF) cells (human embryonic fibroblasts, 3101HUMGNHu33, mycoplasma-free, 80% confluency) were obtained from the Cell Bank of the Shanghai Institutes for Biological Sciences, Chinese Academy of Sciences. THP-1 cells were cultured in RPMI-1640 (Hyclone, Logan) with 10% fetal bovine serum (FBS; Gibco, Gaithersburg) and 0.05 mM β-mercaptoethanol (Sigma, St. Louis). HEF cells were cultured in a modified minimal essential medium (Dulbecco^,^s Modified Eagle Medium [DMEM]) (Hyclone) containing 10% FBS. All cells were maintained in a saturated humidity incubator at 37°C with 5% CO_2_.

The HCMV Towne strain was kindly provided by Professor Jun Fan from the First Affiliated Hospital of Zhejiang University School of Medicine. When HEF cells (5th–6th passage) reached 70% confluency, serum-free DMEM medium was added to the culture flask. The HCMV Towne strain was then inoculated at a multiplicity of infection (MOI) of 2, and the cells were incubated at 37°C in a humidified atmosphere containing 5% CO_2_ for 2 hours. Following the incubation period, the medium was replaced with DMEM containing 2% FBS, and the culture was maintained for an additional 3 to 4 days. Subsequently, HEF cells were harvested, and the supernatant containing HCMV was collected and stored in liquid nitrogen at −80°C.^[10]^

### 2.2. Isolation of single-nucleated cells from peripheral blood and T-cell purification

T cells were isolated from fresh human peripheral blood obtained from healthy donors without HCMV, human immunodeficiency virus, or other common viral infections (hepatitis A, hepatitis B, hepatitis C, hepatitis E, Epstein–Barr virus). The isolation and purification were performed using a human lymphocyte isolation solution (Tianjin Hao Yang Hua Ke Biotechnology Co., Ltd). and a T-cell negative selection magnetic bead sorting kit (No. 11344D; Invitrogen, Carlsbad). The purity of T cells (including CD4^+^ T cells and CD8^+^ T cells) was confirmed by flow cytometry (Leukemia Laboratory), Children’s Hospital, Zhejiang University School of Medicine, BD FACS Calibur), with a purification rate exceeding 85%.

### 2.3. T-cell activation and inhibition

T cells were either activated using phytohemagglutinin (PHA, 5 µg/mL; Roche, Beijing, China) or inhibited by tacrolimus (FK506, 10 ng/ml; Solarbio, Beijing, China) for 12 hours before coculturing with THP-1 cells.^[14,15]^ T cells(at 2 × 10^5^ cells/well) was added to each group except NC2 group.

### 2.4. Establishment of latent infection model and coculture model

#### 2.4.1. Establishment of latent infection model

One day before transfection, THP-1 cells (5th–6th generation cells, cell density 50%–60%) were planted at 2 × 10^5^ cells/well into 2 very low-adherence 6-well plates (Corning, Corning) in a volume of 1 mL culturing medium. On the day of transfection, counted the cells again, 4 × 10^5^ cells per well, added serum-free 1640-diluted lentivirus (MOI = 30) according to the amount of discarded culture medium, and added HCMV Towne at MOI = 5 after 6 hours of culture, and cultured in different conditions to establish a latent infection model.^[10]^ The experiment involved 5 groups (10 well per group): NC1 (THP-1 cells cocultured with untreated T cells), NC2 (HCMV latently infected group without T cells), PHA group (HCMV latently infected group with PHA-activated T cells), FK506 group (HCMV latently infected group with FK506-suppressed T cells), and T-cell group (HCMV latently infected group with untreated T cells). Various parameters were measured at different time points.^[17–19]^ Added T cells (at 2 × 10^5^ cells/well) to each group except the NC2 group on the 6th day of latent infection, then cultured them in RPMI-1640 supplemented with 10% FBS. The experiment was repeated 3 times.

### 2.5. Validation of HCMV latent phase infection

To validate latent HCMV infection in THP-1 cells, we employed a multiparametric approach encompassing cellular morphological analysis, quantitative assessment of intracellular viral genome copies, and evaluation of proliferation-associated gene expression profiles. Viral infection kinetics were monitored at 2 strategically selected time points (5 and 7 days postinfection) to capture critical transition phases. For molecular characterization, we performed simultaneous RNA isolation and protein extraction from HCMV-infected THP-1 cells. The infection status was determined through complementary polymerase chain reaction (PCR) analysis and Western blot detection of 2 key viral proteins: immediate-early protein IE2 (a lytic phase marker) and UL138 (a latent infection indicator), with GAPDH serving as the loading control. The establishment of viral latency was confirmed by the distinctive molecular signature: the absence of IE2 protein expression at 5 days postinfection coupled with sustained UL138 protein detection, which is pathognomonic for the transition to HCMV latent phase.^[10]^

### 2.6. Detection of HCMV DNA by fluorescence quantitative PCR

Fluorescence quantitative PCR (qPCR) was used to detect HCMV DNA in supernatant samples. Sample preparation, reaction mix preparation, and PCR amplification procedures were conducted following standard protocols,^[14]^ with the DNA copy number calculated based on a standard curve.

### 2.7. Reverse transcription qPCR

Four experimental groups (PHA group, T-cell group, FK506 group, and normal control group) were established to investigate the expression of HCMV lncRNA4.9, UL138, RNA extraction, reverse transcription, and PCR amplification were performed using established protocols, and relative expression was analyzed by the 2-ΔΔCT method with GAPDH as the internal control.^[10]^ The primer sequences are presented in Table 1.

**Table 1:** Primer sequences.

Gene names	Sequences
GAPDH	Forward: 5′- GAAGGTGAAGGGTCGGAGTC-3′
	Reverse: 5′-GAAGATGGTGATGGGATTC-3′
lncRNA4.9	Forward: 5′-CCGCCATGACCACCAAAAAG-3′
	Reverse: 5′-GCCGCTCTCTTACGTATCCC-3′
TGF-β1	Forward: 5′-CATACTGGGAATCGTGAAGG-3′
	Reverse: 5′-TTGGACAACGAGAAGGTGC-3′
UL138	Forward: 5′-TATCGTCTGTCCGACTCCCG-3′
	Reverse: 5′-TGGCACGACACCTTCAAACTGG-3′

lncRNA = long noncoding RNA, TGF-β1 = transforming growth factor-β1.

### 2.8. Enzyme-linked immunosorbent assay

Some cell culture supernatant was collected from the 5 groups after 3 and 5 days to detect the protein expression of TGF-β1 by ELISA Kit for transforming-growth factor β1 (Serial. no. 336A99B577; Cloud-Clone Corp., Wuhan, China), the samples were detected with a SynergyH1 multifunction microplate detector (BioTek, Winooski).

### 2.9. Statistical methods

Statistical analyses were performed using SPSS 25.0 (IBM Corp., Armonk) and GraphPad Prism 8.0 (GraphPad Software, San Diego). The normality of data distribution was assessed using the Shapiro–Wilk test. For group comparisons, a one-way analysis of variance was applied for normally distributed data, followed by the Tukey post hoc test for multiple comparisons. Nonnormally distributed data were analyzed using the Kruskal–Wallis test, with the Dunn test for post hoc comparisons. All tests were 2-tailed, and statistical significance was set at *P* < .05.

## 3. Results

### 3.1. Cytokine profiling in the latent infection model

Cytokine expression profiles (IL-2, IL-4, IL-6, and IL-10) were analyzed in the latent infection model using flow cytometry. Cell culture supernatants were collected at 24, 48, and 72 hours post-T-cell coculture. The experimental design comprised 4 distinct groups: normal control group 1 (NC1, THP-1 cells cocultured with untreated T cells), PHA-stimulated group, FK506-treated group, and T-cell-activated group. Normal control group 2 (NC2), consisting of THP-1 cells without T cells, served as a negative control for cytokine and IFN-γ expression.

**Figure 1.**
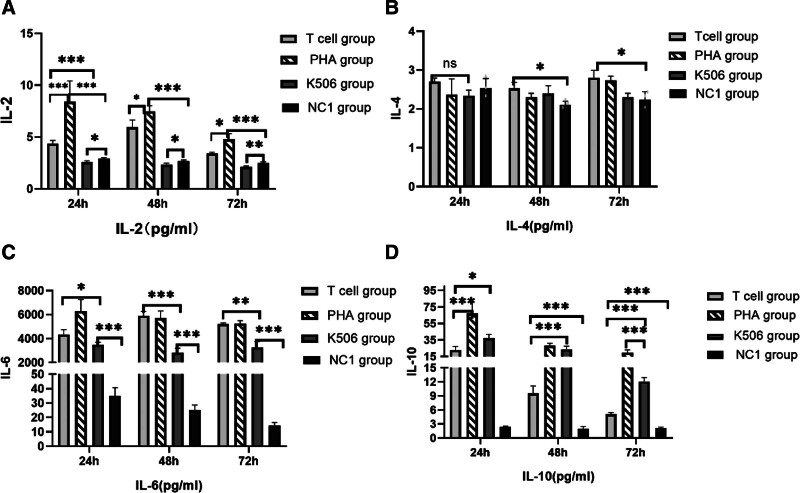
Analysis of T-cell activation by measuring cytokines release. Secretion of (A) IL-2, (B) IL-4, (C) IL-6, (D) IL-10 was detected at 24, 48, and 72 h after T cells added by flow cytometry. PHA. Phytoalexin-activated T-cell group. K506 group. tacrolimus (K506)-suppressed T cells group. NC1 group. normal control group 1. One-way ANOVA was applied for the 2-way comparison between different groups. ***P < .001, **P < .01, *P < .05. ANOVA = analysis of variance, IL = interleukin, PHA = phytohemagglutinin A.

Comparative analysis revealed significant temporal increases in IL-2, IL-6, and IL-10 secretion in both PHA-stimulated and T cell-activated groups relative to NC1 at all time points (Figs. 1A, C, and D). In contrast, IL-4 expression remained stable across all groups, independent of T-cell activation status (Fig. 1B). These findings demonstrate that IL-6 and IL-10 production is specifically upregulated during latent HCMV infection.

### 3.2. Proinflammatory cytokine dynamics in latent infection

**Figure 2.**
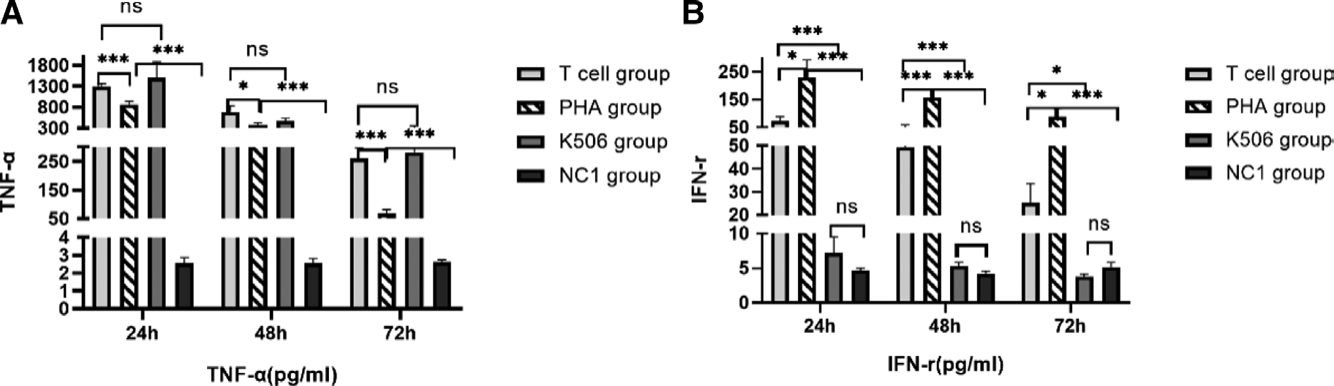
Analysis of T-cell activation by measuring cytokines release. Secretion of TNF-α (A) d IFN-γ (B) was detected at 24, 48, and 72 h after T cells added by flow cytometry. All those groups were compared with the T-cell group. PHA. Phytoalexin-activated T-cell group. K506 group. tacrolimus (K506)-suppressed T cells group. NC 1 group. normal control group 1. One-way ANOVA was applied for the 2-way comparison between different groups. ***P < .001, **P < .01, *P < .05. ANOVA = analysis of variance, IFN-γ = interferon γ, PHA = phytohemagglutinin A, TNF-α = tumor necrosis factor alpha.

The temporal expression patterns of TNF-α and IFN-γ were quantitatively assessed throughout the coculture period. TNF-α secretion demonstrated a progressive increase across all experimental groups compared with NC1 controls (Fig. 2A). Notably, IFN-γ expression was markedly elevated in both T cell-activated and PHA-stimulated groups relative to NC1 (Fig. 2B). These observations establish that TNF-α and IFN-γ pathways are significantly upregulated during latent HCMV infection in the presence of activated T cells.

### 3.3. Molecular markers of latent infection

The expression profiles of lncRNA4.9, UL138, and TGF-β1 were evaluated in 4 experimental conditions: NC2 (HCMV-latently infected THP-1 cells without T cells), PHA-stimulated group, FK506-treated group, and T cell-activated group. NC1 (uninfected THP-1 cells with T cells) served as a negative control for HCMV-specific messenger RNA expression.

**Figure 3.**
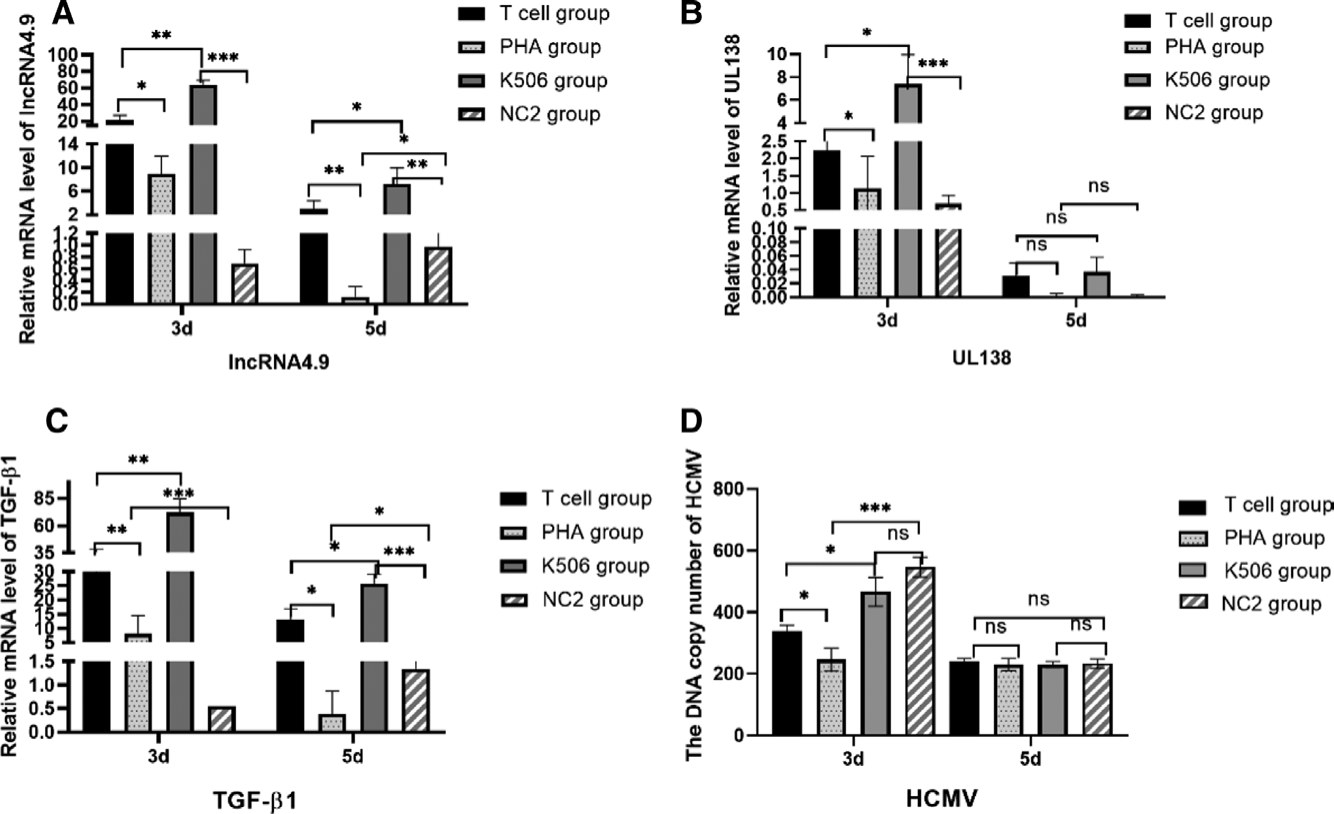
Analysis of RNA and HCMV DNA copy number in the host cells. After 5 day’s treatment, the expression of lncRNA4.9 (A and C), TGF-β1 (B), and UL138 (C) was detected at 3 and 5 d after T cells added by RT-qPCR, the copies of HCMV were detected by qPCR. PHA. Phytoalexin-activated T-cell group. K506 group. tacrolimus (K506)-suppressed T cells group. NC 1 group. normal control group 1.One-way ANOVA was applied for the 2-way comparison between different groups. ***P < .001, **P < .01, *P < .05. ANOVA = analysis of variance, HCMV = human cytomegalovirus, lncRNA = long noncoding RNA, mRNA = messenger RNA, RT-qPCR = reverse transcription-quantitative polymerase chain reaction, TGF-β1 = transforming growth factor-β1.

Quantitative analysis revealed that lncRNA4.9 and TGF-β1 expression levels were significantly elevated in both T cell-activated and FK506-treated groups compared with NC2 following 3 to 5 days of coculture (Fig. 3A–C). The PHA group exhibited a transient increase at day 3, followed by subsequent downregulation. UL138 expression remained stable throughout the 5-day observation period (Fig. 3B). These data suggest that T-cell interaction modulates lncRNA4.9 and TGF-β1 expression during latent infection.

### 3.4. Quantitative analysis of HCMV replication

Viral replication dynamics were assessed through qPCR analysis of HCMV DNA copy number in THP-1 cells following 3 and 5 days coculture periods with differentially treated T cells. Both T cell-activated and PHA-treated groups demonstrated a significant reduction in HCMV DNA copy number at day 3 compared with NC controls (*P* < .05; Fig. 3D). However, this suppressive effect was not maintained at day 5, indicating transient viral suppression during early latent infection.

## 4. Discussion

HCMV infection elicits a robust cell-mediated immune response, characterized by sequential activation of natural killer cells followed by adaptive immune components, including high-affinity neutralizing antibodies, CD4^+^/CD8^+^ T cells, and B cells.^[17–19]^ Despite this comprehensive immune activation, HCMV establishes lifelong persistence through sophisticated immune evasion strategies. The virus encodes cytokine homologs, such as UL146 (IL-8-like) and UL111a (viral IL-10), which play crucial roles in immune modulation.^[20,21]^ Notably, the viral IL-10 homolog suppresses Th1 cytokine production (IFN-γ and IL-2) and inhibits monocyte/macrophage-mediated inflammatory responses while downregulating major histocompatibility complex-II expression, thereby impairing antigen presentation to CD4^+^ T cells.^[22]^

Our experimental findings demonstrate significant upregulation of IL-6, IL-10, and TNF-α in the latent infection model, independent of T-cell activation status. This cytokine profile aligns with previous reports and suggests their potential utility as biomarkers for distinguishing between latent and active HCMV infection states. In immunocompetent individuals, T-cell responses typically maintain effective control over viral reactivation cycles during latent infection.^[23,24]^ HCMV’s ability to establish persistent infection across multiple tissues is facilitated by sustained transcriptional activity of specific viral genes, including UL138, LUNA, US28, UL111A, and UL144.^[25]^

Building upon our previous findings that lncRNA4.9 regulates EGR1 and TGF-β1 expression to promote viral latency,^[11]^ the current study reveals significant upregulation of both lncRNA4.9 and TGF-β1 in T cell and FK506-treated groups compared with NC2 controls. The FK506 group exhibited increased HCMV DNA levels, consistent with previous reports,^[23,24]^ suggesting a temporal correlation between lncRNA4.9/TGF-β1 expression and viral latency establishment.

The immunosuppressive effects of TGF-β are well-documented, particularly its inhibition of T-cell activation and cytokine production (IL-2, IL-10, TNF-α, and IFN-γ).^[16]^ Our temporal analysis revealed distinct expression patterns: PHA treatment induced transient lncRNA4.9 and TGF-β1 upregulation at day 3, followed by progressive downregulation. This pattern correlated with reduced HCMV DNA levels in both T cell and PHA groups, suggesting that PHA-activated T cells effectively suppress viral latency-associated molecules. These findings highlight the potential of lncRNA4.9 and TGF-β1 as quantitative biomarkers for assessing T-cell activation status in HCMV-seropositive individuals.

## 5. Conclusion

This study provides compelling evidence that PHA-activated T cells modulate the lncRNA4.9-TGF-β1 axis during HCMV latent infection. We identified characteristic cytokine profiles (elevated IL-6, IL-10, and TNF-α) associated with viral latency, independent of T-cell activation status. The innovative incorporation of T cells in our experimental model provides novel insights into their regulatory effects on viral latency-associated molecules. While our in vitro findings are promising, we acknowledge the limitation of using cell culture models. Future investigations should prioritize in vivo validation using animal models to strengthen the clinical relevance of these findings.

## Acknowledgments

We thank all the volunteers who participated in the study.

## Author contributions

**Conceptualization:** Xiaolian Yi, Shiqiang Shang.

**Validation:** Xiaolian Yi.

**Visualization:** Xiaolian Yi.

**Writing – original draft:** Xiaolian Yi.

**Writing – review & editing:** Xiaolian Yi, Shiqiang Shang.

**Data curation:** Lifang Liu.

**Formal analysis:** Ran Tao.

**Investigation:** Wei Li.

**Methodology:** Huamei Li.

**Software:** Huamei Li.

**Project administration:** Lin He.

**Resources:** Yujie Liu.
